# How Different Pre-existing Mental Disorders and Their Co-occurrence Affects COVID-19 Clinical Outcomes? A Real-World Data Study in the Southern United States

**DOI:** 10.3389/fpubh.2022.831189

**Published:** 2022-06-16

**Authors:** Shan Qiao, Jiajia Zhang, Shujie Chen, Bankole Olatosi, Suzanne Hardeman, Meera Narasimhan, Larisa Bruner, Abdoulaye Diedhiou, Cheryl Scott, Ali Mansaray, Sharon Weissman, Xiaoming Li

**Affiliations:** ^1^Department of Health Promotion Education and Behavior, South Carolina StateSmart Center for Healthcare Quality, Arnold School of Public Health, University of South Carolina, Columbia, SC, United States; ^2^Department of Epidemiology and Biostatistics, Arnold School of Public Health, University of South Carolina, Columbia, SC, United States; ^3^Department of Health Services Policy and Management, School of Public Health, University of South Carolina, Columbia, SC, United States; ^4^Department of Neuropsychiatry and Behavioral Science, Prisma Health (Midlands), Columbia, SC, United States; ^5^South Carolina Department of Health and Environmental Control, Columbia, SC, United States; ^6^Department of Internal Medicine, School of Medicine, University of South Carolina, Columbia, SC, United States

**Keywords:** pre-existing mental disorders, co-occurrence, COVID-19 outcomes, electronic health records (EHRs), United States

## Abstract

**Background:**

Although a psychiatric history might be an independent risk factor for COVID-19 infection and mortality, no studies have systematically investigated how different clusters of pre-existing mental disorders may affect COVID-19 clinical outcomes or showed how the coexistence of mental disorder clusters is related to COVID-19 clinical outcomes.

**Methods:**

Using a retrospective cohort study design, a total of 476,775 adult patients with lab-confirmed and probable COVID-19 between March 06, 2020 and April 14, 2021 in South Carolina, United States were included in the current study. The electronic health record data of COVID-19 patients were linked to all payer-based claims data through the SC Revenue and Fiscal Affairs Office. Pre-existing mental disorder diagnoses from Jan 2, 2019 to Jan 14, 2021 were extracted from the patients' healthcare utilization data via ICD-10 codes.

**Results:**

There is an elevated risk of COVID-19-related hospitalization and death among participants with pre-existing mental disorders adjusting for key socio-demographic and comorbidity covariates. Co-occurrence of any two clusters was positively associated with COVID-19-related hospitalization and death. The odds ratio of being hospitalized was 1.26 (95% CI: 1.151, 1.383) for patients with internalizing and externalizing disorders, 1.65 (95% CI: 1.298, 2.092) for internalizing and thought disorders, 1.76 (95% CI: 1.217, 2.542) for externalizing and thought disorders, and 1.64 (95% CI: 1.274, 2.118) for three clusters of mental disorders.

**Conclusions:**

Pre-existing internalizing disorders and thought disorders are positively related to COVID-19 hospitalization and death. Co-occurrence of any two clusters of mental disorders have elevated risk of COVID-19-related hospitalization and death compared to those with a single cluster.

## Introduction

The coronavirus disease 2019 (COVID-19) caused by the severe acute respiratory syndrome coronavirus (SARS-CoV-2) has rapidly become a global public health crisis. Individuals with existing mental disorders may be particularly vulnerable to COVID-19 (1–7). A growing body of research focuses on the impact of pre-existing mental disorders on COVID-19 clinical outcomes among this vulnerable population. Pre-existing mental disorders were associated with risk of severe COVID-19 clinical outcomes (hospitalization, intensive care unit [ICU] admission, invasive ventilation, or death) (8–11). Recent studies also found a higher mortality rate among COVID-19 patients with mental disorders compared to the general population (12, 13). Likewise, hospitalized COVID-19 patients with severe psychiatric disorders died at a younger age than those without a psychiatric disorder after controlling for other clinically relevant variables.

Although existing literature suggests that a psychiatric diagnosis might be an independent risk factor for COVID-19 infection and mortality, some knowledge gaps remain. First, no studies have systematically investigated how different clusters of pre-existing mental disorders may affect COVID-19 clinical outcomes (14). Increasing evidence reveals that sets of mental disorders and symptoms predictably co-occur (15, 16). That is, some mental disorders are more highly correlated with each other. Studies about the structure of psychopathology result in the incorporation of disorder clusters in research and the growth of transdiagnostic treatment (17, 18). According to an existing psychopathology structure model (i.e., “three factor model”), mental disorders can be organized into three broad clusters (or higher-order factors): internalizing disorders (e.g., depression, generalized anxiety disorder, panic disorder, and post-traumatic stress disorder), externalizing disorders (e.g., conduct disorder, alcohol dependence, cannabis dependence, other drug dependence, and tobacco addiction), and thought disorders [e.g., obsessive-compulsive disorder (OCD), mania, and schizophrenia] (19–21). Examining the unique roles of these mental disorder clusters in affecting COVID-19 clinical outcomes will advance our understanding of the intersection of mental disorders and infectious diseases in the context of the COVID-19 pandemic.

Second, data are limited on how the co-occurrence of different mental disorder clusters is related to COVID-19 clinical outcomes. Co-occurrence of different mental disorders is not infrequent events. Literature suggests that the rates of co-occurrence of two or more clusters of disorders are very high in psychiatry (e.g., ~50%). That is, about one half of the patients who meet the diagnostic criteria for one disorder in a cluster meet the diagnostic criteria for a second disorder in a different cluster (22). A good understanding of the impact of mental disorders (and their co-occurrences) on COVID-19 outcomes will be important to inform effective surveillance, prevention, and treatment.

Therefore, the current study aims to exam: (1) how each cluster of pre-existing mental disorders (i.e., internalizing disorders, externalizing disorders, and thought disorders) is associated with COVID-19 clinical outcomes; and (2) how the co-occurrences of different mental disorder clusters (i.e., internalizing & externalizing, internalizing & thought, externalizing & thought, internalizing & externalizing & thought) are associated with COVID-19 clinical outcomes. Using population-based electronic health records (EHR) data from a statewide cohort of all confirmed and probable COVID-19 adult cases in South Carolina (SC), United States from March 6, 2020 to April 14, 2021, our study will provide real-world evidence to inform the healthcare delivery for people with pre-existing mental disorders in the context of COVID-19 pandemic and future public health emergencies.

## Methods

### Data Source

Data for this study were extracted from the SC statewide Case Report Form (CRF; “Human Infection With 2019 Novel Coronavirus Case Report Form”) issued by the SC Department of Health and Environmental Control (SC DHEC) (23). Reporting of COVID-19 cases to the SC DHEC is mandated by SC Law and Regulations (24). Specifically, all the key information of COVID-19 cases was collected through the CRF distributed within the SC Infectious Disease and Outbreak Network (SCION), the statewide infectious disease reporting system. The key information of lab-confirmed and probable COVID-19 cases contained in the CRF include the case classification and identification, case demographics, clinical course, symptoms, past medical history, and social history. Hospitalization, ICU and death information were also either provided in the CRF or collected during case interviews by DHEC staff. The criteria of case ascertainment follow the standardized surveillance case definition of COVID-19 (25). The SC Revenue and Fiscal Affairs (RFA) office is the state agency that receives all the medical claims data as required by the state law. The SC RFA office linked the dataset of COVID-19 cases to all medical claim data following our approved research protocol. All data linkage process complied with the Health Insurance Portability and Accountability Act (HIPPA) regulations to protect data privacy and security. After completing the data linkage, the RFA provided the research team with the de-identified dataset for data analysis. The study protocol was approved by the institutional review board at University of South Carolina and SC RFA and SC DHEC who serve as the regulatory bodies of the statewide EHR data. A total of 476,775 adult patients (≥18 years of age) with lab-confirmed and probable COVID-19 between March 06, 2020 and April 14, 2021 were included in the current study.

### Measures

#### Pre-existing Mental Disorders

ICD 10th edition (ICD-10) codes of pre-existing mental disorder diagnoses from January 2, 2019 to January 14, 2021 were extracted from the patients' healthcare utilization data (See Table 1 for ICD-10 codes). A binary variable (yes/no) was created for each of the existing mental disorder clusters. Patients who had diagnosis in least one mental disorder before the COVID-19 diagnosis were defined as “Yes” for this variable and the rest were defined as “No.”

**Table 1:** Summary of ICD-10 codes used for cluster determination.

**Type/cluster**	**Disease name**	**ICD-10 code**
Internalizing disorders	Depression	F32, F33
	Generalized anxiety disorder	F41.1
	Social phobia	F40.1
	Simple phobia	F40.298
	Agoraphobia	F40.0
	Panic disorder	F41.1
	Post-traumatic stress disorder	F43.1
	Eating disorders	F50
Externalizing disorders	Attention-deficit/hyperactivity disorder	F90
	Conduct disorder	F91
	Alcohol dependence	F10.2
	Cannabis dependence	F12.2
	Other drug dependence	F19.2
	Tobacco dependence	F17.2
Thought disorders	Obsessive-compulsive disorder	F42
	Mania	F30
	Schizophrenia	F20–29

#### COVID-19 Clinical Outcomes

Key COVID-19 clinical outcomes included severity, hospitalization, and death. COVID-19 severity was categorized as asymptomatic, mild, and moderate/severe (26). COVID-19 patients who showed no symptoms were categorized as asymptomatic; those who presented any of various mild signs and symptoms of COVID-19 (e.g., fever, cough, sore throat, malaise, headache, muscle pain, nausea, vomiting, diarrhea, loss of taste, and smell) were categorized as mild; and those with difficulty breathing or developed pneumonia or acute respiratory distress syndrome (ARDS) were categorized as moderate/severe. Hospitalization was assessed using the response to the CRF question “Was the patient hospitalized?” (“yes,” “no,” or “unknown”). We then dichotomized the hospitalization (1 = yes, 0 = no or unknown). Death was measured by the response to question “Did the patient die as a result of this illness?” (“yes,” “no,” or “unknown”). It was dichotomized in the same way (1 = death, 0 = alive or unknown).

#### Covariates

Demographic characteristics included age at the time of COVID-19 diagnosis (18–49, ≥50), sex (female, male, and unknown), race (White, Black or African American, Asian, and other/unknown), and ethnicity (Hispanic or Latino, not Hispanic or Latino, and unknown). We created a binary variable of rural/urban residence according to the list of rural counties designated by the Office of Rural Health Policy (27). We employed the Charlson Comorbidity Index (CCI) to evaluate pre-existing comorbidity conditions (28). The CCI scores (equal to 1, 2, 3, or 6) were assigned based on the severity of each condition and derived by adding the scores for all comorbidities in a particular patient. Based on the distribution of CCI score in this cohort, we classified the patients into 3 CCI categories (0, 1, ≥2).We also included smoking status (never smoker, former smoker, current smoker, other/unknown), an established lifestyle factor associated with COVID-19 clinical outcomes as one of covariates in statistical analysis (29).

### Statistical Analysis

Descriptive statistics were computed for all study variables using means and standard deviations (SD) for the continuous variables and frequency counts and percentages for the categorical variables. Differences in key COVID-19 clinical outcomes (i.e., severity, hospitalization, and death) by each mental disorder cluster and covariates were tested using a *t*-test or Chi-square tests as appropriate. A Venn Diagram was created to illustrate patterns of co-occurrence of different clusters of mental disorders among the participants. We examined the association between pre-existing mental disorder cluster and each of the COVID-19 clinical outcomes using logistic regression or multinomial logistic regressions as appropriate separately. Models were adjusted for age, gender, race, ethnicity, residence, smoking status, and CCI score. The models for COVID-19 hospitalization and death were also adjusted for the COVID-19 severity. Adjusted odds ratios (aOR) and their 95% confidence intervals (95% CI) are presented. To investigate the impact of co-occurrence of different mental disorder clusters, we further examined the three-way interactions among three clusters of mental disorders in the final regression models. Forest plots were created based on the final regression models to visualize the impacts to COVID-19 clinical outcomes by all the factors including pre-existing mental disorders, co-occurrence of different mental disorder clusters, and other covariates. It is notable that not all the COVID-19 cases included in the current study have been linked to the medical claim data. Only the individuals who had visited healthcare service system in SC reported health insurance status and had other pre-exiting medical records. Therefore, we ran additional subgroup analysis for the individuals with health visits and had insurance information and compared the results with the ones based on whole group. All the analyses were conducted using SAS, version 9.4.

## Results

### Characteristics of Participants

Of the 476,775 adult COVID-19 patients included in the study, 421,475 patients had no recorded clinical diagnosis of a mental disorder and 55,300 patients had at least one diagnosis of mental disorders before the COVID-19 diagnosis (Table 2). Among the overall sample, 58.3% were 18–49 years of age (mean = 45.7, SD = 18.6), 52.9% were female, 47.6% were White, 20.4% were Black, 5.1% were Hispanic/Latino, and the majority were living in urban areas (87.0%). Over one half (54.0%) of the patients were identified as asymptomatic cases, 33.3% as mild cases, and 12.7% as moderate/severe cases. About 4.4% of all patients (*n* = 20,995) were hospitalized and 1.9% (*n* = 8,924) died due to COVID-19.

**Table 2:** Characteristics of the whole study population.

**Characteristics**	**Overall population**	**Any mental disorder cluster**		**Internalizing disorders**		**Externalizing disorders**		**Thought disorders**	
		**No**	**Yes**	**No**	**Yes**	**No**	**Yes**	**No**	**Yes**
**Age**									
18-49	277,988 (58.31)	249,969 (59.31)	28,019 (50.67)	263,832 (59.46)	14,156 (42.78)	259,275 (58.1)	18,713 (61.27)	276,914 (58.4)	1,074 (41.09)
50+	198,787 (41.69)	171,506 (40.69)	27,281 (49.33)	179,849 (40.54)	18,938 (57.22)	186,960 (41.9)	11,827 (38.73)	197,247 (41.6)	1,540 (58.91)
**Gender**									
Female	252,292 (52.92)	219,867 (52.17)	32,425 (58.63)	229,550 (51.74)	22,742 (68.72)	237,537 (53.23)	14,755 (48.31)	251,014 (52.94)	1,278 (48.89)
Male	208,956 (43.83)	187,738 (44.54)	21,218 (38.37)	199,609 (44.99)	9,347 (28.24)	194,059 (43.49)	14,897 (48.78)	207,712 (43.81)	1,244 (47.59)
Unknown/missing	15,527 (3.26)	13,870 (3.29)	1,657 (3)	1,4522 (3.27)	1,005 (3.04)	14,639 (3.28)	888 (2.91)	15,435 (3.26)	92 (3.52)
**Race**									
White	226,985 (47.61)	200,400 (47.55)	26,585 (48.07)	208,767 (47.05)	18,218 (55.05)	213,968 (47.95)	13,017 (42.62)	225,950 (47.65)	1,035 (39.59)
Black	97,066 (20.36)	82,522 (19.58)	14,544 (26.3)	90,289 (20.35)	6,777 (20.48)	87,672 (19.65)	9,394 (30.76)	96,192 (20.29)	874 (33.44)
Other/Unknown	149,240 (31.3)	135,179 (32.07)	14,061 (25.43)	141,213 (31.83)	8,027 (24.26)	141,162 (31.63)	8,078 (26.45)	148,541 (31.33)	699 (26.74)
**Ethnicity**									
Not hispanic or latino	285,738 (59.93)	250,038 (59.32)	35,700 (64.56)	263,844 (59.47)	21,894 (66.16)	266,520 (59.73)	19,218 (62.93)	284,126 (59.92)	1,612 (61.67)
Hispanic or Latino	24,526 (5.14)	23,260 (5.52)	1,266 (2.29)	23,780 (5.36)	746 (2.25)	23,839 (5.34)	687 (2.25)	24,497 (5.17)	29 (1.11)
Unknown/missing	166,511 (34.92)	148,177 (35.16)	18334 (33.15)	156,057 (35.17)	10,454 (31.59)	155,876 (34.93)	10,635 (34.82)	165,538 (34.91)	973 (37.22)
**Residence**									
Rural	61,816 (12.97)	53,448 (12.68)	8,368 (15.13)	57,133 (12.88)	4,683 (14.15)	56,944 (12.76)	4,872 (15.95)	61,432 (12.96)	384 (14.69)
Urban	414,959 (87.03)	368,027 (87.32)	46,932 (84.87)	386,548 (87.12)	28,411 (85.85)	389,291 (87.24)	25,668 (84.05)	412,729 (87.04)	2,230 (85.31)
**Smoking**									
No	71,451 (14.99)	61,684 (14.64)	9,767 (17.66)	64,363 (14.51)	7,088 (21.42)	67,559 (15.14)	3,892 (12.74)	70,994 (14.97)	457 (17.48)
Former smoker	17,274 (3.62)	14,392 (3.41)	2,882 (5.21)	15,445 (3.48)	1,29 (5.53)	15,724 (3.52)	1,550 (5.08)	17,207 (3.63)	67 (2.56)
Current smoker	8,417 (1.77)	5,408 (1.28)	3,009 (5.44)	7,521 (1.7)	896 (2.71)	5,580 (1.25)	2,837 (9.29)	8,325 (1.76)	92 (3.52)
Other/Unknown	379,633 (79.63)	339,991 (80.67)	39,642 (71.69)	356,352 (80.32)	23,281 (70.35)	357,372 (80.09)	22,261 (72.89)	377,635 (79.64)	1,998 (76.43)
**CCI score**									
**0**	375,705 (78.8)	356,688 (84.63)	19,017 (34.39)	367,602 (82.85)	8,103 (24.48)	362,533 (81.24)	13,172 (43.13)	375,110 (79.11)	595 (22.76)
**1**	28,531 (5.98)	19,721 (4.68)	8,810 (15.93)	23,235 (5.24)	5,296 (16.00)	23,559 (5.28)	4,972 (16.28)	28,189 (5.95)	342 (13.08)
**≥2**	72,539 (15.21)	45,066 (10.69)	27,473 (49.68)	52,844 (11.91)	19,695 (59.51)	60,143 (13.48)	12,396 (40.59)	70,62 (14.94)	1,677 (64.15)
**COVID severity/syms**									
No/asymptomatic	257,254 (53.96)	226,574 (53.76)	30,680 (55.48)	239,324 (53.94)	17,930 (54.18)	239,666 (53.71)	17,588 (57.59)	255,553 (53.9)	1,701 (65.07)
Mild	158,960 (33.34)	143,639 (34.08)	15,321 (27.71)	150,046 (33.82)	8,914 (26.94)	150,623 (33.75)	8,337 (27.3)	158,395 (33.41)	565 (21.61)
Severe	60,561 (12.7)	51,262 (12.16)	9,299 (16.82)	54,311 (12.24)	6,250 (18.89)	55,946 (12.54)	4,615 (15.11)	60,213 (12.7)	348 (13.31)
**COVID hospitalization**									
No	455,780 (95.6)	405,328 (96.17)	50,452 (91.23)	426,194 (96.06)	29,586 (89.4)	427,289 (95.75)	28,491 (93.29)	453,527 (95.65)	2,253 (86.19)
Yes	20,995 (4.4)	16147 (3.83)	4,848 (8.77)	17,487 (3.94)	3,508 (10.6)	18,946 (4.25)	2,049 (6.71)	20,634 (4.35)	361 (13.81)
**Death**									
No	467,851 (98.13)	414,714 (98.4)	53,137 (96.09)	436,379 (98.35)	31,472 (95.1)	438,099 (98.18)	29,752 (97.42)	465,416 (98.16)	2,435 (93.15)
Yes	8,924 (1.87)	6,761 (1.6)	2,163 (3.91)	7,302 (1.65)	1,622 (4.9)	8,136 (1.82)	788 (2.58)	8,745 (1.84)	179 (6.85)

*The results for Asian group were not reported due to policy. Chi-square tests for all the characteristics variables are all significant (i.e., P < 0.05)*.

### Pre-existing Mental Disorders

**Figure 1:**
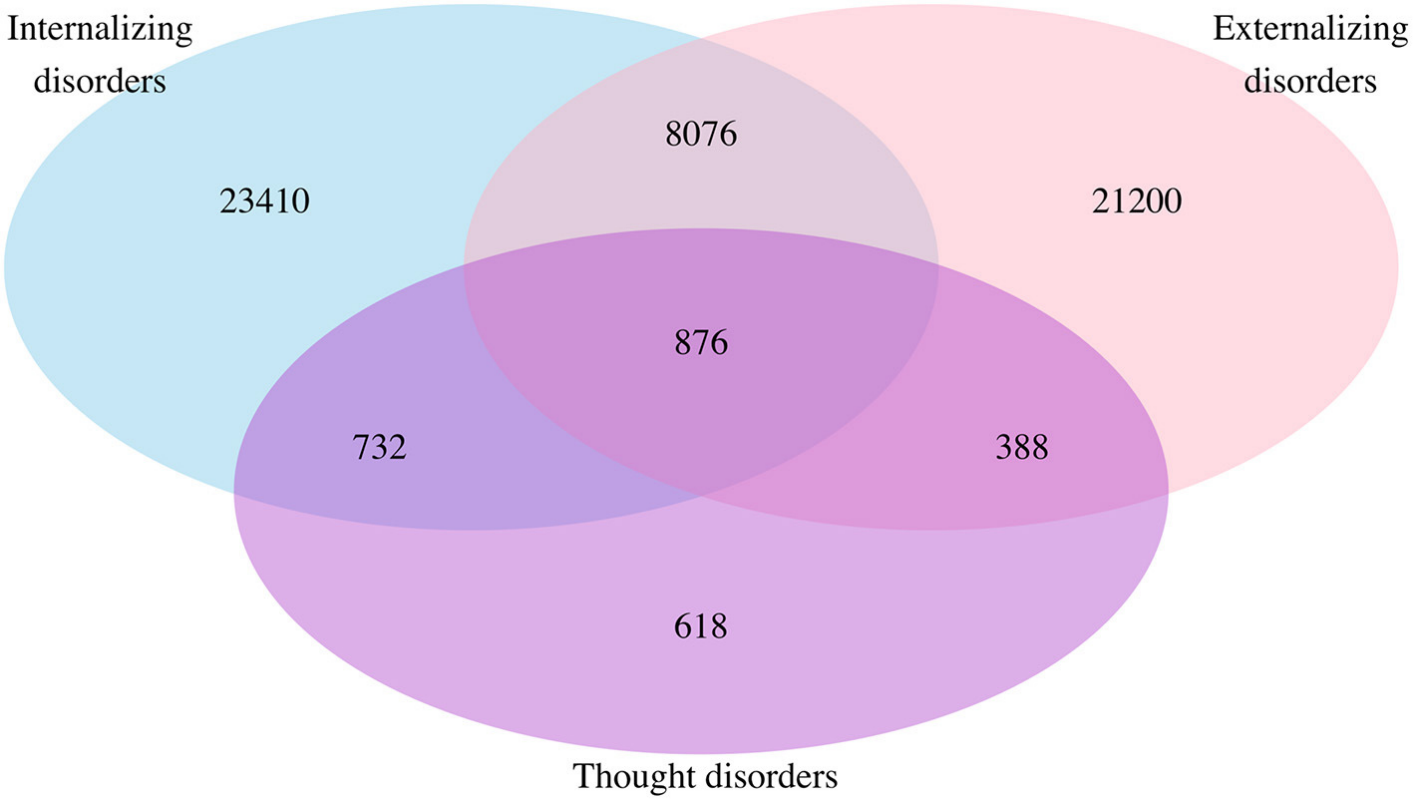
Venn diagram for comorbidity of mental disorders among the whole population: Sample in each mental disorder cluster. Not any mental disorders = 421,475; internalizing disorders only = 23,410; externalizing disorders only = 21,200; thought disorders only = 618; internalizing and externalizing disorders = 8,076; internalizing and thought disorders = 732; externalizing and thought disorders = 388; internalizing, externalizing, and thought disorders = 876.

Of the 55,300 patients with pre-existing mental disorders, 23,410 (42.3%) had internalizing disorders only, 21,200 (38.3%) had externalizing disorders only, and 618 (1.1%) had thought disorders only. The number of individuals with a single cluster or multiple clusters of mental disorders is presented in Figure 1. There were 8,076 (14.6%) patients who had both internalizing and externalizing disorders, 732 (13.2%) had both internalizing and thought disorders, and 388 (0.7%) had externalizing and thought disorders. The number of patients having all three clusters of internalizing, externalizing, and thought disorders was 876 (1.6%).

Generally, we observed significant differences in age, gender, race, ethnicity, residence, smoking status, and pre-existing comorbidity conditions between participants with and without pre-existing mental disorders (Table 2). For example, there was a higher proportion of people who were older, female, non-Hispanic/Latino, living in rural area and with comorbidity conditions among participants with pre-existing mental disorders, compared with those without these disorders. However, the patterns of the socio-demographic difference between participants with and without pre-existing mental disorders varied by the mental disorder clusters. Higher proportions of participants with internalizing disorders were older (≥50 years), female, White, non-smokers, and lived in rural areas. Higher proportions of participants with externalizing disorders were males, Black/African American, former/ current smokers, and lived in rural areas. We found that higher proportions of participants with thought disorders were older (≥50 years), Black/African American, non-smokers, and lived in rural areas.

### COVID-19 Clinical Outcomes

Among the 55,300 patients who had at least one pre-existing mental disorder, 8.8% (*n* = 4,848) were hospitalized and 3.9% (*n* = 2,163) died due to COVID-19. Compared to those without respective mental disorders, participants with pre-existing mental disorders have a higher proportion of COVID-19 hospitalization (Internalizing disorders: 10.6 vs. 3.9%; Externalizing disorders: 6.7 vs. 4.3%; Thought disorders: 13.8 vs. 4.4%; Any mental disorder cluster: 8.8 vs. 3.8%) and COVID-19-related deaths (Internalizing disorders: 4.9 vs. 1.7%; Externalizing disorders: 2.6 vs. 1.8%; Thought disorders: 6.9 vs. 1.8%; Any mental disorder cluster: 3.9 vs. 1.6%). The association between pre-existing mental disorders and COVID-19 severity was more complicated. In general, participants with pre-existing mental disorders had higher proportions of being asymptomatic (Internalizing disorders: 54.2 vs. 53.9%; Externalizing disorders: 57.6 vs. 53.7%; Thought disorders: 65.1 vs. 53.9%; Any cluster of mental disorders: 55.5 vs. 53.8%) and severe cases (Internalizing disorders: 18.9 vs. 12.2%; Externalizing disorders: 15.1 vs. 12.5%; Thought disorders: 13.3 vs. 12.7%; Any mental disorder cluster: 16.8 vs. 12.2%). However, they represented a lower proportion of mild cases (Internalizing disorders: 26.9 vs. 33.8%; Externalizing disorders: 27.3 vs. 33.8%; Thought disorders: 21.6 vs. 33.4%; Any mental disorder cluster: 27.7 vs. 34.1%).

### Multivariate Analysis

Adjusting for key socio-demographic and comorbidity covariates (i.e., age, gender, race, ethnicity, residence, smoking, comorbidity), the multivariate results suggest an elevated risk of COVID-19-related hospitalization and death among participants with pre-existing mental disorders except externalizing disorders (Table 3). For COVID-19 hospitalization, the adjusted OR of having internalizing disorders, externalizing disorders, and thought disorders was 1.19 (95% CI: 1.130, 1.258), 0.931 (95% CI: 0.867, 1.000), and 2.39 (95% CI: 1.899, 3.006), respectively. Similarly, for COVID-19-related death, the aOR of having internalizing disorders, externalizing disorders, and thought disorders was 1.13 (95% CI: 1.056, 1.214), 0.775 (95% CI: 0.697, 0.860), and 2.34 (95% CI: 1.789, 3.052), respectively. However, pre-existing mental disorders were associated with decreased risk of mild and severe cases. Specifically, having internalizing disorders (aOR = 0.85, 95% CI: 0.818, 0.884), externalizing disorders (aOR = 0.74, 95% CI: 0.708, 0.765), and thought disorders (aOR = 0.72, 95% CI: 0.575, 0.903) were negatively associated with mild cases. Having externalizing disorders (aOR = 0.77; 95% CI: 0.730, 0.807) was negatively associated with severe cases but having internalizing disorders (aOR = 1.10; 95% CI: 1.051, 1.151) was positively associated with severe cases.

**Table 3:** Regression model results for COVID-19 outcomes adjusting for covariates in the whole study population.

**Items**	**Severity (mild vs. no) aOR (95%CI)**	**Severity (severe vs. no) aOR (95%CI)**	**Hospitalization aOR (95%CI)**	**Death aOR (95%CI)**
Age 50+ vs. 18–49	**0, 711 (0.700, 0.723)**	**0.719 (0.703, 0.735)**	**6.171 (5.919, 6.435)**	**21.189 (19.015, 23.611)**
Gender male vs. female	**0.909 (0.895, 0.923)**	**0.828 (0.811, 0.845)**	**1.511 (1.464, 1.560)**	**1.567 (1.497, 1.639)**
Gender unknown/missing vs. Female	**0.916 (0.871, 0.963)**	**0.848 (0.789, 0.911)**	**0.762 (0.672, 0.865)**	**0.731 (0.610, 0.877)**
Race black vs. white	**0.820 (0.805, 0.836)**	**0.862 (0.841, 0.883)**	**1.839 (1.776, 1.906)**	**1.169 (1.109, 1.232)**
Race Asian vs. white	1.041 (0.963, 1.124)	0.983 (0.881, 1.097)	**1.528 (1.264, 1.848)**	1.262 (0.931, 1.710)
Race other/unknown vs. white	**0.253 (0.247, 0.259)**	**0.264 (0.254, 0.273)**	**0.868 (0.818, 0.921)**	**0.834 (0.770, 0.902)**
Ethnicity hispanic or latino vs. not hispanic or latino	**2.048 (1.979, 2.120)**	**2.449 (2.341, 2.563)**	**1.582 (1.462, 1.711)**	0.961 (0.833, 1.108)
Ethnicity unknown/missing vs. not hispanic or latino	**0.291 (0.285, 0.297)**	**0.277 (0.268, 0.285)**	**0.675 (0.642, 0.711)**	**0.877 (0.820, 0.939)**
Urban vs. rural	**1.050 (1.027, 1.074)**	**1.097 (1.065, 1.130)**	**0.905 (0.868, 0.944)**	**0.842 (0.794, 0.892)**
Current smoker vs. No	**0.899 (0.833, 0.971)**	1.020 (0.940, 1.107)	**0.594 (0.531, 0.666)**	**0.603 (0.500, 0.726)**
Former smoker vs. No	**1.478 (1.385, 1.578)**	**2.001 (1.870, 2.140)**	**0.892 (0.841, 0.947)**	**0.691 (0.632, 0.757)**
Other/Unknown vs. No	**0.096 (0.094, 0.099)**	**0.065 (0.063, 0.067)**	**0.931 (0.896, 0.967)**	1.018 (0.963, 1.076)
CCI 1 vs. 0	**0.808 (0.782, 0.835)**	**1.113 (1.068, 1.160)**	**1.559 (1.462, 1.663)**	**1.691 (1.532, 1.866)**
CCI ≥2 vs. 0	**0.555 (0.541, 0.569)**	**0.940 (0.911, 0.969)**	**3.566 (3.437, 3.699)**	**3.969 (3.770, 4.179)**
Internalizing disorders Yes vs. No	**0.850 (0.818, 0.884)**	**1.100 (1.051, 1.151)**	**1.192 (1.130, 1.258)**	**1.132 (1.056, 1.214)**
Externalizing disorders Yes vs. No	**0.736 (0.708, 0.765)**	**0.767 (0.730, 0.807)**	0.931 (0.867, 1.000)	**0.775 (0.697, 0.860)**
Thought disorders Yes vs. No	**0.720 (0.575, 0.903)**	0.768 (0.579, 1.017)	**2.389 (1.899, 3.006)**	**2.336 (1.789, 3.052)**
Internal & external vs. No	**0.577 (0.541, 0.615)**	**0.889 (0.826, 0.956)**	**1.262 (1.151, 1.383)**	0.976 (0.858, 1.110)
Internal & thoughts vs. No	**0.699 (0.568, 0.860)**	0.981 (0.776, 1.241)	**1.648 (1.298, 2.092)**	**1.509 (1.125, 2.023)**
External & thoughts vs. No	**0.562 (0.421, 0.749)**	**0.497 (0.335, 0.737)**	**1.759 (1.217, 2.542)**	**1.874 (1.222, 2.876)**
Internal & external & thought vs. No	**0.399 (0.326, 0.489)**	**0.496 (0.389, 0.631)**	**1.643 (1.274, 2.118)**	0.779 (0.514, 1.181)
Symptom mild vs. asymptomatic	**N/A**	**N/A**	**1.407 (1.343, 1.475)**	**0.859 (0.802, 0.920)**
Symptom severe vs. asymptomatic	**N/A**	**N/A**	**10.744 (10.285, 11.224)**	**4.887 (4.604, 5.187)**

*Bold OR indicate P < 0.05*.

**Figure 2:**
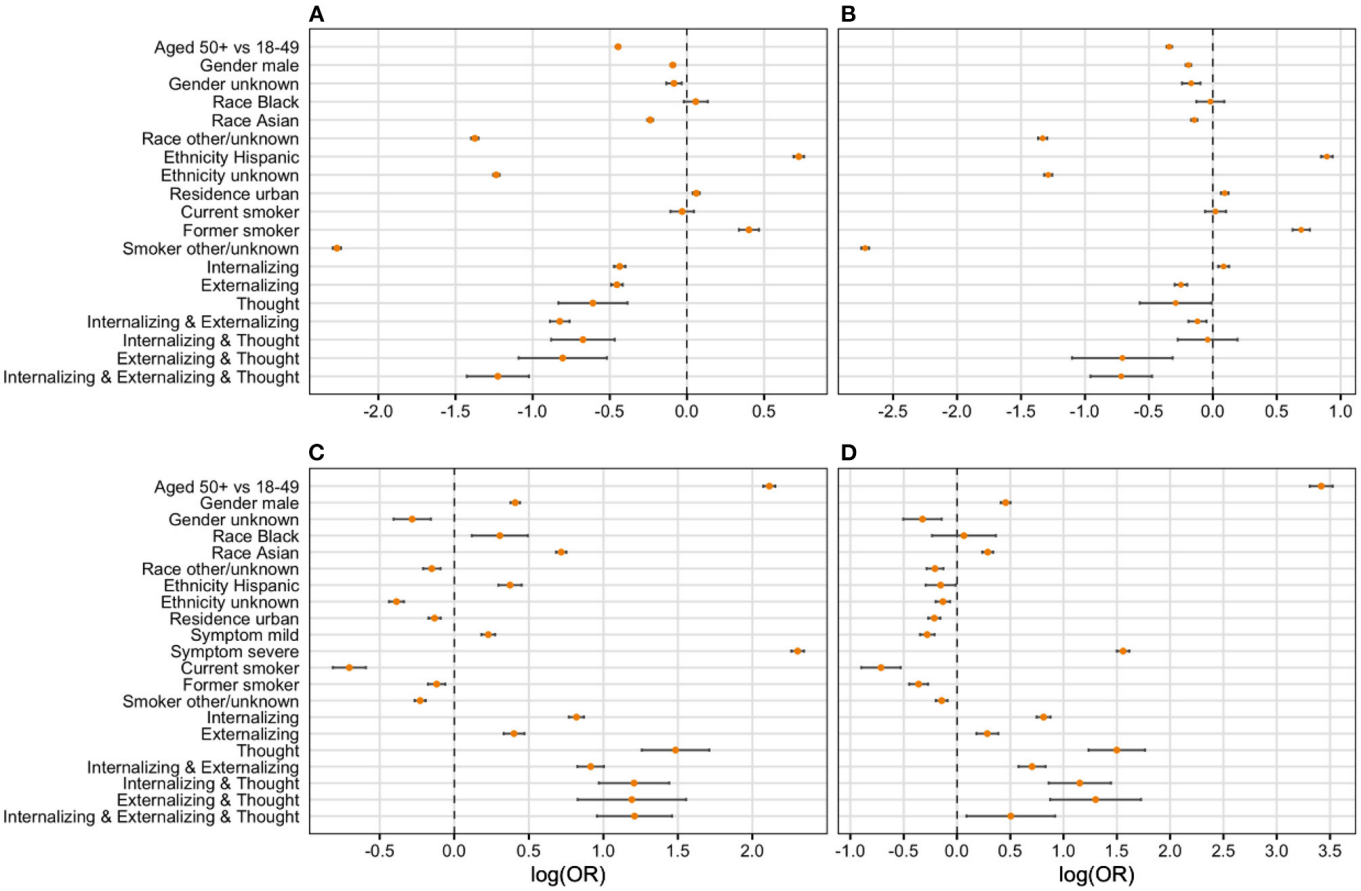
Forest plot of final regression models. Logarithm of odds ratio was used in developing the forest plots given large value of some odds ratios. We then use zero instead of one as the criteria of significance. **(A)** Severity (mild vs. asymp); **(B)** severity (severe vs. asymp); **(C)** hospitalization (yes vs. no); **(D)** death (yes vs. no).

Co-occurrence of any two clusters was positively associated with COVID-19 hospitalization and COVID-19-related death (See the forest plot in Figure 2 and Table 3). Specifically, the odds of being hospitalized was 26% greater among patients with the presence of both internalizing and externalizing disorders (aOR = 1.26; 95% CI: 1.151, 1.383) than the odds of being hospitalized among those with no pre-existing mental disorders. We have 95% confidence that the true parameter falls between 1.151 and 1.383. The odds of being hospitalized was 65% greater among patients with internalizing and thought disorders (aOR = 1.65; 95% CI: 1.298, 2.092), 76% greater among patients with externalizing and thought disorders (aOR = 1.76; 95% CI: 1.217, 2.542), and 64% greater among patients with three clusters of mental disorders (aOR = 1.64; 95% CI: 1.274, 2.118) than the odds of being hospitalized among patients without pre-existing mental disorders. Similarly, the odds of having COVID-19-related death were 51% greater among patients with internalizing and thought disorders (aOR = 1.51, 95% CI: 1.125, 2.023) and 87% greater among patients with externalizing and thought disorders (aOR = 1.87, 95% CI: 1.222, 2.876) than the odds of having COVID-19-related death among patients without pre-existing mental health. Co-occurrence of any two clusters was negatively related with mild cases. The odds of having mild symptoms decreased 42% among patients with internalizing and externalizing disorders (aOR = 0.58, 95% CI: 0.541, 0.615), 30% for patients with internalizing and thoughts disorders (aOR = 0.70, 95% CI: 0.568, 0.860), 44% for patients with externalizing and thoughts disorders (aOR = 0.56, 95% CI: 0.421, 0.749), and 60% for patients with three clusters of mental orders (aOR = 0.40, 95% CI: 0.326, 0.489) than the odds of having mild symptoms among patients without pre-existing mental disorders. In addition, the odds of having severe symptoms decreased by 11% for patients with internalizing and externalizing disorders (aOR = 0.89, 95% CI: 0.826, 0.956), by 50% among patients with externalizing and thought disorders (aOR = 0.50, 95% CI: 0.335, 0.737), and by 50% for patients with three clusters of mental disorders (aOR = 0.50, 95% CI: 0.389, 0.631) than the odds of having severe symptoms among patients without pre-existing mental disorders.

### Subgroup Analysis

The results of subgroup analysis are presented in supplemental tables (Appendix 1). In summary, the subgroup with health visits (*n* = 221,288) was similar in terms of demographic characteristics as the whole sample except older (50^+^ group: 51.6 vs. 41.7%) with the uninsurance rate of 11.1%, which is close to the 12.7% in general population in SC. Multivariate analysis among the subgroup were comparable to the ones based on whole group.

## Discussion

Using a psychopathology structure model of mental disorders (i.e., the three-factor model) and leveraging the EHR data from a statewide cohort of COVID-19 patients in SC, United States, we explored the association between pre-existing mental disorders and COVID-19 clinic outcomes from a new perspective. Our findings are aligned with existing evidence of a positive association between pre-existing mental disorders and COVID-19 related hospitalization and death. Several studies have posited potential reasons and interpretations regarding the underlying mechanism of pre-existing mental disorders as a risk factor of COVID-19 clinical outcomes, including immune dysregulation processes, genetic predisposition toward psychiatric disorders, and health-compromising lifestyle (20). The activation of the endocrine stress axis at different levels, such as hypothalamic-pituitary-adrenal (HPA) axis which can lead to altered glucocorticoids and suppressed cell-mediated and humoral immunity is widely reported among people with mental disorders (30–33). The immune dysfunction, subsequently, contributes to high risk of SARS-CoV-2 infection. The inflammatory cytokine overproduction induced by glucocorticoid receptor resistance may be responsible for the damage of the lungs and correlates with disease deterioration and fatal COVID-19 (34–36). Recent studies also indicate a genetic association between psychiatric disorder and COVID-19 (37). For example, one genome-wide association study suggests that genetic liability to depression and substance misuse is associated with severe COVID-19 outcomes (hospitalization or death) (38).

Internalizing disorders, like depression and anxiety disorders have been found to be associated with immune-inflammatory disturbances, which may contribute to severe COVID-19 clinical outcomes (39, 40). The lifestyle changes following the occurrence of internalizing disorders, such as smoking and alcohol abuse, may also contribute to altered risk to COVID-19 infection and worse outcomes. Externalizing disorders, especially long-term use of tobacco, alcohol, and other drugs, are also closely related to cardiovascular, pulmonary, and metabolic diseases, which are high-risk preconditions for COVID-19 outcomes (41, 42). In addition, we discovered that pre-existing thought disorders (e.g., mania, schizophrenia, OCD) may be related to COVID-19 hospitalization and death in the greatest magnitude.

Thought disorders are usually considered as severe mental disorders. A recent systematic review and meta-analysis on the association between mental disorders and COVID-19 death reports that people with severe mental disorders (defined as schizophrenia and/or bipolar disorders) have the highest risk of COVID-19 death (14). One national-level study conducted in South Korea suggests that severe mental disorders (defined as non-affective or affective disorders with psychotic features) are more likely to be associated with severe clinical outcomes (ICU admission, invasive ventilation, death) compared to other mental disorders (10). People with thought disorders likely need assistance and support from caregivers to follow healthcare and COVID-19 mitigation measures such as mask wearing and social distancing. Some prevention recommendations, such as hand washing, can reinforce the irrational beliefs of patients with OCD (43). The limited availability of caregivers and tailored COVID-19 mitigation strategies during the pandemic makes this group especially vulnerable to COVID-19. In addition, the pre-existing disadvantages at multiple socioecological levels among patients with severe mental disorders have exacerbated the risks of COVID-related hospitalization and mortality. For example, people with schizophrenia are often heavy smokers because nicotine may temporarily lessen the symptoms of poor concentration, low mood, and stress (44–46). They smoke at two to four times the rate of the general population (47). Smokers with a mental health disorder tend to smoke more cigarettes than those in the general population (47). People with severe mental illness also suffer from substantial weight-gain because of metabolic syndrome and psychotropic agents, including antipsychotic medications and antidepressants (48, 49). The higher prevalence rates of cigarette smoking, chronic obstructive pulmonary disease, overweight, hyper-glycaemia/diabetes, hypertension, and cardiovascular disease among people with serious mental illness compared to general population may directly increase their risk of COVID-19 mortality (50). Beside the lifestyle and physical problems, social/structural level factors such as poverty, stigma and discrimination against mental health, social isolation and loneliness also worsen their life circumstances and impede them access to social support and timely/quality healthcare services, resulting in severe COVID-19-related clinical outcomes (51, 52).

We found that people with co-occurrence of any two clusters of mental disorders have elevated risk of COVID-19-related hospitalization and death compared to those with a single cluster (e.g., either internalizing or externalizing disorder). Given that having pre-existing thought disorders is a high-risk factor of worse COVID-19 clinical outcomes, it is reasonable to expect that co-occurrence of thought disorders and either internalizing or externalizing disorders indicates more vulnerability compared to the single cluster of pre-existing mental disorders. Notably, the co-occurrence of internalizing and externalizing disorders has increased risk of COVID-19-related hospitalization compared to a single cluster.

Although our results suggest that pre-existing mental disorders are risk factors of COVID-19-related hospitalization and death, pre-existing mental disorders may be associated with decreased risk of mild cases. Extant literature suggests that pre-existing chronic illness and therapeutic options may affect the prognosis of COVID-19 and have a possible protective effect against COVID-19. For example, allergic sensitization in asthma is related to lower expression of angiotensin-converting enzyme 2 receptors showing a potential protective effect (53). In addition, the use of inhaled corticosteroids is generally safe and associated with decreased risk of hospitalization (54). However, there is a dearth of evidence regarding whether mental disorder treatments affect prognosis of COVID-19. Given the high rate of comorbidity between mental disorders and chronic somatic disease, the pre-existing chronic conditions and the treatment among these patients could be complicated. Further studies are needed to identify potential confounders and explain the “protective effect” (54). In addition, the high-risk drug-drug interactions that may occur in COVID-19 treatment accompanied by psychotropic drug prescription warrants multidisciplinary study engaging both psychiatrists and infectious disease physicians (55).

Our findings need to be interpreted with caution due to several limitations. First, our mental health data comes from a health utilization dataset. Therefore, we were not able to retrieve information of people who had mental disorders but failed to access to healthcare system for any reasons. There is also missing information in the COVID-19 data. The missing data may impede the robustness of our findings. Second, the study is subject to common limitations of using ICD10 codes to define mental health disorders. As other EHR based studies suggest, the quality of raw data may influence the validity of our results. Fourth, we did not differentiate the severity category of the mental disorders. For example, we did not explore if patients with “acute” disorders have any different risk of worse COVID-19 outcomes compared to those with “recurrent” (more severe mental illness) disorders. Fifth, the EHR data is not able to cover multiple socio-economic factor variables or socio-behavioral variables, which could be critical risk factors of COVID-19 clinical outcomes. Finally, the theoretical frame of clustering the mental disorders needs more empirical evidence for further modification and refinement.

Despite these limitations, the current study sheds lights on impacts of different mental disorder clusters and their co-occurrences on COVID-19 clinical outcomes using a large statewide and real-world dataset. Our findings have significant implications for improving surveillance and triage in COVID-19 treatment considering a high prevalence of psychiatric disorders (20.6% as of 2019) in the general population (56). First, it is critical to identify vulnerable subgroups in COVID-19 treatment. Pre-existing thought disorders and co-occurrence of multiple mental disorder clusters seem to be independent risk factors of COVID-19-related hospitalization and death controlling other background characteristics. Identifications of these risk factors will assist triage and medical resource allocation so that the healthcare providers can identify the most vulnerable subgroup and prevent the disease deterioration among them.

Second, sociodemographic profiles of patients with comorbidity of both COVID-19 and mental disorders warrant further exploration. Our findings show that higher proportions of patients with thought disorders were older (≥50 years), Black/African American, and lived in rural areas. Mounting evidence indicates mental health disparities in the United States with racial/ethnic minorities and people living in rural areas being disadvantaged in terms of medical and mental health care resources. Furthermore, this group faces higher risk of COVID-19 infection, endures more barriers to access to health resources, and shows disproportionally high morbidity and mortality of COVID-19. More empirical evidence and policy studies are needed to illustrate and address the intersection between mental health, COVID-19, and social deprivation/vulnerability from the perspective of health equity and structural racism.

## Conclusion

The current study suggests that individuals with mental disorders, especially thought disorders and co-occurrence of multiple mental disorder clusters should been identified as high-risk population for severe consequences of COVID-19, requiring enhanced preventive, triage, and treatment strategies. Future studies need to further differentiate the impacts of mental disorders on COVID-19 clinical outcomes by severity/stage of the disorders (e.g., acute vs. stabilized); include a comprehensive set of social determinates of health in the analysis; and explore the interaction between different mental disorder clusters and pre-existing somatic conditions.

## Data Availability Statement

The University of South Carolina is prohibited from making individual level data available publicly due to provisions in our data use agreements with state agencies/data providers, institutional policy, and ethical requirements. In order to facilitate research, we make access to such data available via approved data access requests. Some USC data is not available externally or for re-release due to prohibitions in data use agreements with our state agencies or other data providers. Institutional policies stipulate that all external requests for data access require collaboration with a (author's affiliation) researcher. For more information or to make a request, please contact (BO): olatosi@mailbox.sc.edu. The underlying analytical codes are available from the authors on request.

## Ethics Statement

The studies involving human participants were reviewed and approved by University of South Carolina. Written informed consent for participation was not required for this study in accordance with the national legislation and the institutional requirements.

## Author Contributions

SQ, JZ, and XL contributed to the study conception and design. Material preparation and data analysis were performed by JZ and SC. The first draft of the manuscript was written by SQ. All authors reviewed and revised the manuscript and read and approved the final manuscript.

## Funding

Research reported in this publication was supported by the National Institutes of Health under Award #R01AI127203-5S1.

## Author Disclaimer

The content is solely the responsibility of the authors and does not necessarily represent the official views of the National Institutes of Health.

## Conflict of Interest

The authors declare that the research was conducted in the absence of any commercial or financial relationships that could be construed as a potential conflict of interest.

## Publisher's Note

All claims expressed in this article are solely those of the authors and do not necessarily represent those of their affiliated organizations, or those of the publisher, the editors and the reviewers. Any product that may be evaluated in this article, or claim that may be made by its manufacturer, is not guaranteed or endorsed by the publisher.
