# Supplementary material for: How Different Pre-existing Mental Disorders and Their Co-occurrence Affects COVID-19 Clinical Outcomes? A Real-World Data Study in the Southern United States
Source: Front Public Health. 2022 Jun 16;10:831189. doi: 10.3389/fpubh.2022.831189 (PMC9244141; doi:10.3389/fpubh.2022.831189)
Supplement: Supplementary file 1 [file Data_Sheet_1.pdf]

Appendix Table 1. Characteristics of the subgroup with healthcare visit

Characteristics	Overall population	Any Mental Disorder Cluster		Internalizing Disorders		Externalizing Disorders		Thought Disorders	
		No	Yes	No	Yes	No	Yes	No	Yes
**Age**									
18-49	107,202 (48.44)	79,183 (47.7)	28,019 (50.67)	93,046 (49.44)	14,156 (42.78)	88,489 (46.39)	18,713 (61.27)	106,128 (48.53)	1,074 (41.09)
50+	114,086 (51.56)	86,805 (52.3)	27,281 (49.33)	95,148 (50.56)	18,938 (57.22)	102,259 (53.61)	11,827 (38.73)	112,546 (51.47)	1,540 (58.91)
**Gender**									
Female	128,252 (57.96)	95,827 (57.73)	32,425 (58.63)	105,510 (56.06)	22,742 (68.72)	113,497 (59.5)	14,755 (48.31)	126,974 (58.07)	1,278 (48.89)
Male	86,421 (39.05)	65,203 (39.28)	21,218 (38.37)	77,074 (40.95)	9,347 (28.24)	71,524 (37.5)	14,897 (48.78)	85,177 (38.95)	1,244 (47.59)
Unknown/missing	6,615 (2.99)	4,958 (2.99)	1,657 (3)	5,610 (2.98)	1,005 (3.04)	5,727 (3)	888 (2.91)	6,523 (2.98)	92 (3.52)
**Race**									
White	101,684 (45.95)	75,099 (45.24)	26,585 (48.07)	83,466 (44.35)	18,218 (55.05)	88,667 (46.48)	13,017 (42.62)	100,649 (46.03)	1,035 (39.59)
Black	59,182 (26.74)	44,638 (26.89)	14,544 (26.3)	52,405 (27.85)	6,777 (20.48)	49,788 (26.1)	9,394 (30.76)	58,308 (26.66)	874 (33.44)

Characteristics	Overall population	Any Mental Disorder Cluster		Internalizing Disorders		Externalizing Disorders		Thought Disorders	
		No	Yes	No	Yes	No	Yes	No	Yes
Other/Unknown	59,551 (26.91)	45,490 (27.41)	14,061 (25.43)	51,524 (27.38)	8,027 (24.26)	51,473 (26.98)	8,078 (26.45)	58,852 (26.91)	699 (26.74)
**Ethnicity**									
Not Hispanic or Latino	141,393 (63.9)	105,693 (63.68)	35,700 (64.56)	119,499 (63.5)	21,894 (66.16)	122,175 (64.05)	19,218 (62.93)	139,781 (63.92)	1,612 (61.67)
Hispanic or Latino	7,841 (3.54)	6,575 (3.96)	1,266 (2.29)	7,095 (3.77)	746 (2.25)	7,154 (3.75)	687 (2.25)	7,812 (3.57)	29 (1.11)
Unknown/missing	72,054 (32.56)	53,720 (32.36)	18,334 (33.15)	61,600 (32.73)	10,454 (31.59)	61,419 (32.2)	10,635 (34.82)	71,081 (32.51)	973 (37.22)
**Residence**									
Rural	34,502 (15.59)	26,134 (15.74)	8,368 (15.13)	29,819 (15.84)	4,683 (14.15)	29,630 (15.53)	4,872 (15.95)	34,118 (15.6)	384 (14.69)
Urban	186,786 (84.41)	139,854 (84.26)	46,932 (84.87)	158,375 (84.16)	28,411 (85.85)	161,118 (84.47)	25,668 (84.05)	184,556 (84.4)	2,230 (85.31)
**Smoking**									
No	42,289 (19.11)	32,522 (19.59)	9,767 (17.66)	35,201 (18.7)	7,088 (21.42)	38,397 (20.13)	3,892 (12.74)	41,832 (19.13)	457 (17.48)

Characteristics	Overall population	Any Mental Disorder Cluster		Internalizing Disorders		Externalizing Disorders		Thought Disorders	
		No	Yes	No	Yes	No	Yes	No	Yes
Former smoker	10,119 (4.57)	7,237 (4.36)	2,882 (5.21)	8,290 (4.41)	1,829 (5.53)	8,569 (4.49)	1,550 (5.08)	10,052 (4.6)	67 (2.56)
Current smoker	4,762 (2.15)	1,753 (1.06)	3,009 (5.44)	3,866 (2.05)	896 (2.71)	1,925 (1.01)	2,837 (9.29)	4,670 (2.14)	92 (3.52)
Other/Unknown	164,118 (74.16)	124,476 (74.99)	39,642 (71.69)	140,837 (74.84)	23,281 (70.35)	141,857 (74.37)	22,261 (72.89)	162,120 (74.14)	1,998 (76.43)
**CCI Score**									
0	120,218 (54.33)	101,201 (60.97)	19,017 (34.39)	112,115 (59.57)	8,103 (24.48)	107,046 (56.12)	13,172 (43.13)	119,623 (54.7)	595 (22.76)
1	28,531 (12.89)	19,721 (11.88)	8,810 (15.93)	23,235 (12.35)	5,296 (16)	23,559 (12.35)	4,972 (16.28)	28,189 (12.89)	342 (13.08)
>=2	72,539 (32.78)	45,066 (27.15)	27,473 (49.68)	52,844 (28.08)	19,695 (59.51)	60,143 (31.53)	12,396 (40.59)	70,862 (32.41)	1,677 (64.15)
**Insurance status**									
Uninsured	24,611 (11.12)	16,354 (9.85)	8,257 (14.93)	21,781 (11.57)	2,830 (8.55)	17,899 (9.38)	6,712 (21.98)	24,213 (11.07)	398 (15.23)
Insured	196,544 (88.82)	149,530 (90.08)	47,014 (85.02)	166,294 (88.36)	30,250 (91.41)	172,735 (90.56)	23,809 (77.96)	194,329 (88.87)	2,215 (84.74)

Characteristics	Overall population	Any Mental Disorder Cluster		Internalizing Disorders		Externalizing Disorders		Thought Disorders	
		No	Yes	No	Yes	No	Yes	No	Yes
Unknown	133 (0.06)	104 (0.06)	29 (0.05)	119 (0.06)	14 (0.04)	114 (0.06)	19 (0.06)	132 (0.06)	
**COVID Severity/Syms**									
No/asymptomatic	116,137 (52.48)	85,457 (51.48)	30,680 (55.48)	98,207 (52.18)	17,930 (54.18)	98,549 (51.66)	17,588 (57.59)	114,436 (52.33)	1,701 (65.07)
Mild	71,116 (32.14)	55,795 (33.61)	15,321 (27.71)	62,202 (33.05)	8,914 (26.94)	62,779 (32.91)	8,337 (27.3)	70,551 (32.26)	565 (21.61)
Severe	34,035 (15.38)	24,736 (14.9)	9,299 (16.82)	27,785 (14.76)	6,250 (18.89)	29,420 (15.42)	4,615 (15.11)	33,687 (15.41)	348 (13.31)
**COVID Hospitalization**									
No	205,825 (93.01)	155,373 (93.6)	50,452 (91.23)	176,239 (93.65)	29,586 (89.4)	177,334 (92.97)	28,491 (93.29)	203,572 (93.09)	2,253 (86.19)
Yes	15,463 (6.99)	10,615 (6.4)	4,848 (8.77)	11,955 (6.35)	3,508 (10.6)	13,414 (7.03)	2,049 (6.71)	15,102 (6.91)	361 (13.81)
**Death**									

Characteristics	Overall population	Any Mental Disorder Cluster		Internalizing Disorders		Externalizing Disorders		Thought Disorders	
		No	Yes	No	Yes	No	Yes	No	Yes
No	214,506 (96.94)	161,369 (97.22)	53,137 (96.09)	183,034 (97.26)	31,472 (95.1)	184,754 (96.86)	29,752 (97.42)	212,071 (96.98)	2,435 (93.15)
Yes	6,782 (3.06)	4,619 (2.78)	2,163 (3.91)	5,160 (2.74)	1,622 (4.9)	5,994 (3.14)	788 (2.58)	6,603 (3.02)	179 (6.85)

Note: The results for Asian group and some frequency of people with unknown insurance status were not reported due to SC DHEC policy of reporting frequency <10. Chi-square tests for all the characteristics variables are all significant (i.e., P<.05).

Appendix Table 2. Regression model results for COVID-19 outcomes adjusting for covariates in the subgroup with healthcare visit

Items	Severity (mild vs no)	Severity (severe vs no)	Hospitalization	Death
	aOR (95%CI)	aOR (95%CI)	aOR (95%CI)	aOR (95%CI)
Age 50+ vs 18-49	**0.710 (0.692,0.728)**	**0.708 (0.686,0.731)**	**4.711 (4.470,4.965)**	**13.993 (12.273,15.954)**
Gender Male vs Female	**0.909 (0.888,0.930)**	**0.865 (0.840,0.891)**	**1.488 (1.432,1.546)**	**1.590 (1.509,1.676)**
Gender Unknown/missing vs Female	1.006 (0.931,1.086)	0.959 (0.867,1.061)	**0.719 (0.617,0.839)**	**0.737 (0.594,0.914)**
Race Black vs White	**0.899 (0.876,0.923)**	**0.908 (0.879,0.937)**	**1.636 (1.569,1.705)**	**1.084 (1.022,1.151)**
Race Asian vs White	1.140 (0.970,1.341)	1.092 (0.888,1.343)	**1.884 (1.441,2.462)**	1.185 (0.768,1.828)
Race Other/Unknown vs White	**0.272 (0.262,0.283)**	**0.272 (0.258,0.287)**	**0.810 (0.753,0.872)**	**0.806 (0.733,0.886)**
Ethnicity Hispanic or Latino vs Not Hispanic or Latino	**2.222 (2.091,2.362)**	**2.698 (2.506,2.905)**	**1.409 (1.262,1.574)**	0.823 (0.674,1.004)
Ethnicity Unknown/missing vs Not Hispanic or Latino	**0.306 (0.297,0.316)**	**0.285 (0.273,0.298)**	**0.656 (0.617,0.697)**	**0.830 (0.766,0.899)**

**Items**	**Severity (mild vs no)**	**Severity (severe vs no)**	**Hospitalization**	**Death**
	**aOR (95%CI)**	**aOR (95%CI)**	**aOR (95%CI)**	**aOR (95%CI)**
Urban vs Rural	**1.065 (1.032,1.098)**	**1.104 (1.062,1.147)**	**0.913 (0.869,0.958)**	**0.855 (0.801,0.914)**
Current smoker vs No	0.980 (0.885,1.086)	**1.198 (1.076,1.333)**	**0.630 (0.553,0.719)**	**0.650 (0.526,0.803)**
Former smoker vs No	**1.513 (1.391,1.646)**	**2.113 (1.939,2.302)**	0.935 (0.873,1.001)	**0.704 (0.636,0.779)**
Other/Unknown vs No	**0.089 (0.086,0.092)**	**0.067 (0.065,0.070)**	**1.056 (1.009,1.104)**	**1.133 (1.062,1.208)**
CCI 1 vs 0	**0.789 (0.762,0.817)**	1.010 (0.967,1.055)	**1.557 (1.452,1.669)**	**2.022 (1.808,2.262)**
CCI >=2 vs 0	**0.534 (0.518,0.549)**	**0.860 (0.830,0.891)**	**3.747 (3.576,3.926)**	**4.848 (4.498,5.225)**
Insured vs Uninsured	**1.147 (1.105,1.190)**	1.001 (0.955,1.049)	1.045 (0.969,1.127)	**1.677 (1.45,1.939)**
Unknown vs Uninsured	1.023 (0.662,1.580)	0.925 (0.532,1.610)	0.746 (0.337,1.650)	**3.114 (1.355,7.160)**
Internalizing disorders Yes vs No	**0.855 (0.822,0.889)**	**1.070 (1.022,1.120)**	**1.162 (1.102,1.226)**	**1.131 (1.055,1.213)**
Externalizing disorders Yes vs No	**0.738 (0.709,0.768)**	**0.703 (0.667,0.740)**	**0.912 (0.848,0.980)**	**0.802 (0.721,0.891)**

Items	Severity (mild vs no)	Severity (severe vs no)	Hospitalization	Death
	aOR (95%CI)	aOR (95%CI)	aOR (95%CI)	aOR (95%CI)
Thought disorders Yes vs No	**0.713 (0.569,0.895)**	**0.736 (0.556,0.974)**	**2.387 (1.899,3.00)**	**2.359 (1.805,3.082)**
Internal & external Yes vs No	**0.583 (0.546,0.623)**	**0.845 (0.785,0.909)**	**1.204 (1.099,1.319)**	0.976 (0.858,1.111)
Internal & thought Yes vs No	**0.703 (0.571,0.865)**	0.956 (0.757,1.207)	**1.598 (1.262,2.022)**	**1.497 (1.117,2.005)**
External & thought Yes vs No	**0.563 (0.422,0.752)**	**0.459 (0.310,0.681)**	**1.749 (1.215,2.517)**	**1.928 (1.256,2.958)**
Internal &External &Thought Yes vs No	**0.403 (0.329,0.494)**	**0.469 (0.368,0.597)**	**1.586 (1.234,2.039)**	0.791 (0.522,1.199)
Symptom Mild vs Asymptomatic	**n/a**	**n/a**	**1.588 (1.502,1.679)**	0.970 (0.896,1.050)
Symptom Severe vs Asymptomatic	**n/a**	**n/a**	**10.551 (10.015,11.116)**	**4.912 (4.584,5.263)**

Note: Bold OR indicate P-value <.05.

Appendix Figure 1 Forest plot of final regression models

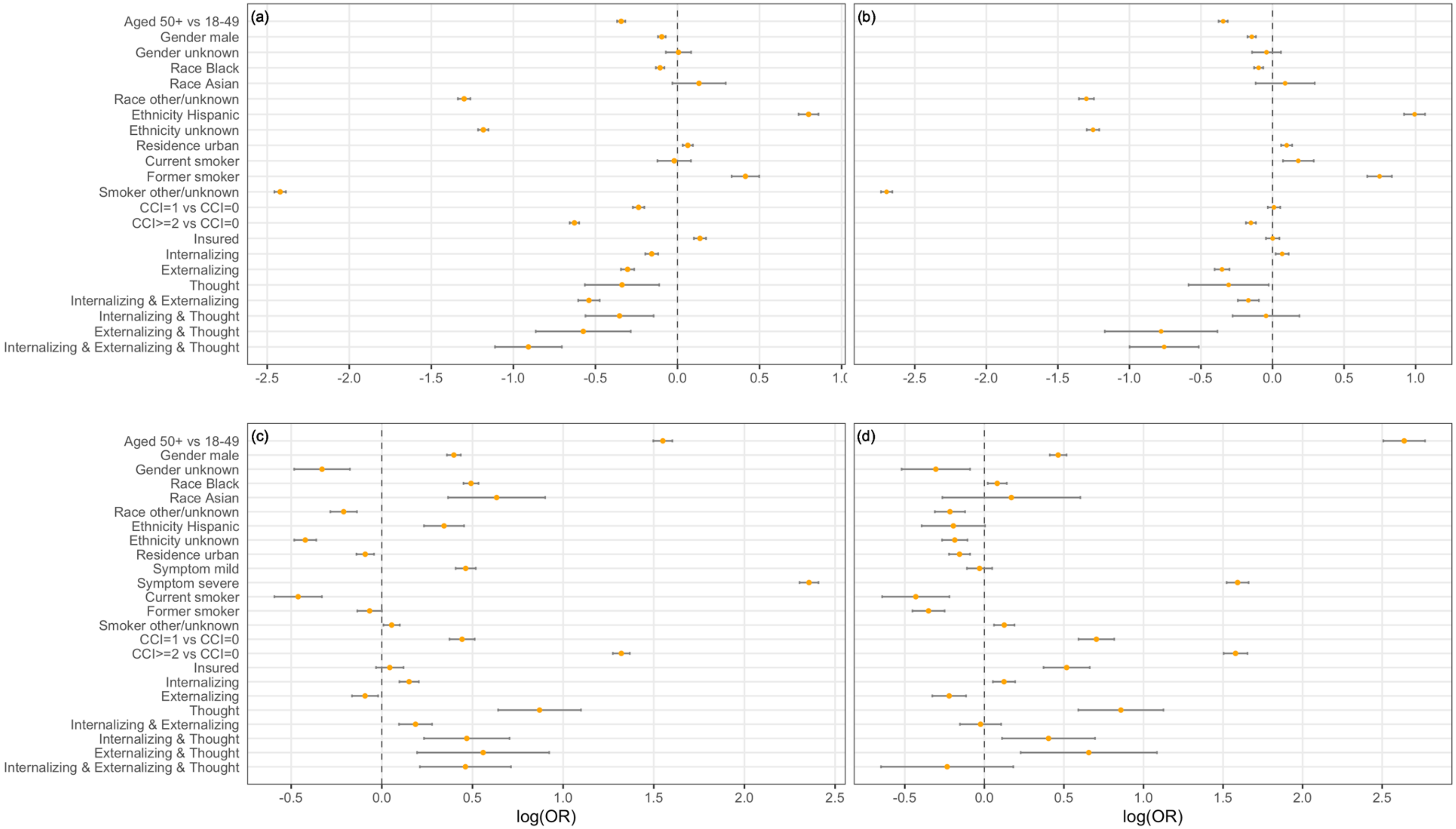

Note: Logarithm of odds ratio was used in developing the forest plots given large value of some odds ratios. We then use zero instead of one as the criteria of significance. (a) severity (mild vs asymp); (b) severity (severe vs asymp); (c) hospitalization (yes vs no); (d) death (yes vs no).
